# Tobacco use prevalence – disentangling associations between Alaska Native race, low socio-economic status and rural disparities

**DOI:** 10.3402/ijch.v72i0.21582

**Published:** 2013-08-05

**Authors:** Julia A. Dilley, Erin Peterson, Matthew Bobo, Kathryn E. Pickle, Kristen Rohde

**Affiliations:** 1 Program Design and Evaluation Services, Multnomah County Health Department and Oregon Health Authority, Portland, OR, USA; 2 Alaska Department of Health and Social Services, Anchorage, AK, USA

**Keywords:** Alaska/epidemiology, Smoking/epidemiology, Prevalence, Smoking/ethnology, Indians, North American, Tobacco, smokeless

## Abstract

**Background:**

Tobacco use rates are exceptionally high among indigenous people in North America. Alaska Native, low socio-economic status (SES) and rural communities are high-priority populations for Alaska's Tobacco Control program.

**Design:**

For the purpose of better informing tobacco control interventions, we conducted a descriptive study to describe high-priority groups using prevalence-based and proportion-based approaches.

**Methods:**

With data from 22,311 adults interviewed for Alaska's 2006–2010 Behavioral Risk Factor Surveillance System (BRFSS), we used stratified analysis and logistic regression models to describe the current use of cigarettes and smokeless tobacco (SLT) (including iq'mik, a unique Alaska Native SLT product) among the 3 populations of interest.

**Results:**

“Population segments” were created with combinations of responses for Alaska Native race, SES and community type. We identified the highest prevalence and highest proportion of tobacco users for each type of tobacco by “segment”. For cigarette smoking, while the largest proportion (nearly one-third) of the state's smokers are non-Native, high SES and live in urban settings, this group also has lower smoking prevalence than most other groups. Alaska Native, low SES, rural residents had both high smoking prevalence (48%) and represented a large proportion of the state's smokers (nearly 10%). Patterns were similar for SLT, with non-Native high-SES urban residents making up the largest proportion of users despite lower prevalence, and Alaska Native, low SES, rural residents having high prevalence and making up a large proportion of users. For iq'mik use, Alaska Native people in rural settings were both the highest prevalence and proportion of users.

**Conclusion:**

While Alaska Native race, low SES status and community of residence can be considered alone when developing tobacco control interventions, creating “population segments” based on combinations of factors may be helpful for tailoring effective tobacco control strategies and messaging. Other countries or states may use a similar approach for describing and prioritizing populations.

Tobacco use remains the leading cause of preventable death and disease in the United States and worldwide (1). With each passing year, additional information has become available that describes the health consequences of using tobacco (2); yet, despite this evidence, individuals continue to purchase and use tobacco products. Some recent studies have in particular documented extremely high tobacco use among circumpolar indigenous populations. For example, Chateau-Degat et al. measured 84% smoking among Inuit adults in Quebec (3); Egeland, Cao and Young in a separate study measured 69% smoking among Inuit adults across Canada (4); and the State of Alaska has documented smoking prevalence among Alaska Native adults in excess of 40%, approximately double that of the non-Native population (5).

One reason that tobacco continues to be so widely used worldwide is the sophisticated promotion and other marketing approaches used by the tobacco industry, which spent more than $8.5 billion on cigarette and smokeless tobacco (SLT) marketing in 2010 for the United States alone (6). Although the tobacco industry has far more resources than public health, spending more than $18 to market tobacco products for every $1 spent by states in the United States to reduce tobacco use (7), some approaches used by the industry to promote tobacco products can be studied and replicated at relatively low cost for the purpose of reducing tobacco use.

Marketing science has long recognized the value of identifying “market segments,” or profiles of potential customer clusters, who can then be the focus of advertising messages (8). Segmentation is still widely used to market diverse products and ideas (9). Yet, most public health programs describe priority populations only in single terms and underutilize the approach of marketing segmentation to refine and focus delivery of messages to “population segments.”

The State of Alaska has successfully implemented a tobacco control effort since 1996 and has observed significant reductions in both adult and youth tobacco use in the general population (10). Alaska Native communities and people with lower socio-economic status (low SES) are examples of high-priority population groups for Alaska's tobacco control efforts, due to continued high tobacco use prevalence (11). Tobacco use is also higher outside the relatively few densely populated areas in the state, such as Anchorage and Fairbanks (12). These 3 factors (Alaska Native race, low SES status and rural location) are often correlated; for example, Alaska Native people are more likely than non-Natives in Alaska to live in rural communities, and also to be lower income (13). Other factors may also be important to consider when targeting anti-tobacco messages, such as age, employment status and whether children are present in the home.

With multiple possible combinations of demographic characteristics that could be considered, leading to potentially unmanageable numbers of “population segments” for targeted intervention, it may also be useful to consider how to prioritize those groups. Prioritization of the largest population groups for interventions may be appealing because of the potential to significantly change statewide prevalence estimates. However, failure to identify and appropriately serve high-prevalence subgroups is likely to aggravate health inequities and is a failure if the program's mandate is to serve an entire population. Both approaches for identifying priority populations have merit, and approaching prioritization from both lenses can provide the clearest picture of need when planning. Identifying high-priority “segments” may also help to monitor the effect of interventions in different groups, as a contribution to efforts to decrease health disparities.

The purpose of this analysis is to define and describe “high-priority population segments” for tobacco control, based both on prevalence of use and proportions of users and to understand more about their characteristics. We used a market segmentation-like approach to describe different clusters of adult current smokers and current SLT users in the State of Alaska, specifically examining characteristics of Alaska Native race, low SES and urban–rural residence because of their priority for the state program and implications for program design. Findings may be useful for informing tobacco control interventions, including marketing of tobacco cessation messages or cultural adaptation of interventions in the state, and may also provide an example for other states or countries working to deliver tobacco control effectively in diverse community settings with indigenous populations.

## Methods

### Data sources

We used data from 22,311 adults interviewed as part of the 2006–2010 Alaska Behavioral Risk Factor Surveillance System (BRFSS). BRFSS is a CDC-sponsored statewide, random-digit-dial landline telephone survey of adults (people older than 18 years). The survey collects information about a variety of demographic and health factors. Alaska's BRFSS is administered only in English. Alaska's BRFSS methods and variables used in this study are consistent with federal guidelines (14).

### Measures

#### Outcomes: tobacco use

A standard BRFSS definition of current smoking status was used (having smoked at least 100 cigarettes during a lifetime, and currently smoking “every day” or “some days”). We categorized the smoking outcome as “current” vs. “non-current” use. Current SLT users were similarly classified as “current” for respondents who answered “yes” to the question “Do you currently use any SLT products such as chewing tobacco, snuff, iq'mik or Blackbull?” Current iq'mik users were SLT users who specifically reported using iq'mik (a unique SLT variant prevalent in Southwest Alaska) (15).

#### Demographics

Respondents provided their exact age and highest level of formal education completed. They also provided information about total household income (estimated) and whether there were children in the home. Employment status was classified based on self-report as: currently employed (including self-employed), unemployed or not in the workforce (homemaker, student, retired or unable to work).

Individuals were classified as “lower SES” if they had less than a high-school education *or* an income <185% of Alaska's federally determined poverty level.

Alaska Native race was defined based on self-reported single race or preferred race as “American Indian or Alaska Native” (AIAN). We use the term “Alaska Native” in this article, as do most reports and documents for the state, because most AIAN people in Alaska are of Alaska Native heritage (13).

Urban–hub–rural community residence was determined based on federal classifications using a self-reported home zipcode and telephone prefix. Only the city of Anchorage, the immediate surrounding area, and the city of Fairbanks qualified as “urban” or metropolitan areas. “Hub” (micropolitan) included areas such as municipalities of Juneau and Bethel, which are off the road system but serve as gateways and provide centralized services to smaller villages that surround them. “Rural” areas included hundreds of villages throughout the state, often with only 100–500 residents.

### Analysis

Data were weighted to adjust for sampling design (based on region and telephone listing), and for the number of telephones and adults in each household. Data were also post-stratified to the age and sex distribution of the Alaska State population. Procedures took into account complex sampling design and post-stratification weights. We used Stata 10.1^®^ for all analyses.

We used logistic regression models to examine tobacco use prevalence among specific groups, both stratified and adjusted for multiple demographic factors. We used the Stata “svytotal” command to estimate population sizes; this procedure takes into account the weights applied to individual observations in the sample to calculate total numbers in a population.

We created 12 “population segments” that represented all combinations of these variables (Alaska Native race: yes/no; lower SES: yes/no; and community type: urban, hub, rural), and used stratified analysis to describe their tobacco use.

## Results

Tobacco use prevalence for cigarettes, all SLT and iq'mik alone are shown overall and for demographic subgroups in Table I. Cigarette smoking prevalence was 21.3% for the state overall. After adjustment for all other demographic characteristics, most of the identified factors remained significantly associated with higher odds of smoking including male gender, younger age, being unemployed, lower SES or Alaska Native race. Living in hub or rural community settings and having children in the home were not significantly associated with increased odds for smoking after adjustment for other factors.

**Table I:** Tobacco use prevalence and odds for tobacco use in specific demographic groups

	Cigarette smoking			Smokeless tobacco use			Iq'mik use		
			
Alaska BRFSS 2006–2010 combined	% Smoking (95% CI)	Crude odds ratio (95% CI)	Adjusted odds ratio (95% CI)	% use (95% CI)	Crude odds ratio (95% CI)	Adjusted* odds ratio (95% CI)	% use (95% CI)	Crude odds ratio (95% CI)	Adjusted* odds ratio (95% CI)
Total (n=22,311)	21.3 (20.4–22.2)	–	–	4.9 (4.5–5.3)	–	–	0.7 (0.6–0.8)	–	–
Gender: Female (n=12,181)	19.5 (18.5–20.6)	Referent	Referent	1.4 (1.1–1.6)	Referent	Referent	0.8 (0.6–1.0)	Referent	Referent
Gender: Male (n=10,130)	23 (21.6–24.4)	1.2 (1.1–1.4)	1.2 (1.1–1.4)	8.2 (7.4–9.0)	(5.3–8.0)	7.0 (5.6–8.6)	0.6 (0.5–0.8)	0.7 (0.5–1.0)	0.7 (0.5–0.9)
Age: 50 or better (n=10,437)	16.1 (15.2–17.2)	Referent	Referent	2.7 (2.3–3.1)	Referent	Referent	0.5 (0.4–0.7)	Referent	Referent
Age: 30–49 (n=8,579)	21.2 (20.0–22.5)	1.4 (1.3–1.6)	1.4 (1.2–1.6)	6.2 (5.5–7.0)	2.4 (1.9–2.9)	2.0 (1.6–2.6)	0.8 (0.6–1.0)	1.6 (1.1–2.3)	1.1 (0.7–1.7)
Age: 18–29 (n=3,007)	29.7 (27.1–32.4)	2.2 (1.9–2.5)	1.8 (1.6–2.1)	6.1 (5.0–7.4)	2.3 (1.8–3.0)	1.9 (1.4–2.5)	0.8 (0.6–1.2)	1.7 (1.0–2.7)	0.9 (0.6–1.6)
No children at home (n=13,216)	19.6 (18.5–20.7)	Referent	Referent	3.6 (3.2–4.1)	Referent	Referent	0.3 (0.2–0.4)	Referent	Referent
Children at home (n=9,033)	23.3 (21.9–24.8)	1.2 (1.1–1.4)	0.9 (0.8–1.0)	6.3 (5.6–7.1)	1.8 (1.5–2.2)	1.3 (1.0–1.6)	1.2 (1.0–1.4)	4.6 (3.0–6.9)	2.1 (1.3–3.2)
Currently employed (n=14,542)	19.5 (18.4–20.5)	Referent	Referent	5.2 (4.7–5.8)	Referent	Referent	0.5 (0.4–0.6)	Referent	Referent
Currently unemployed (n=1,565)	45.0 (40.9–49.1)	3.4 (2.8–4.1)	2.0 (1.7–2.5)	10.6 (8.5–13.0)	2.1 (1.7–2.8)	1.2 (0.9–1.7)	3.1 (2.3–4.2)	6.6 (4.5–9.7)	1.6 (1.0–2.3)
Currently not in workforce (n=6.066)	19.4 (17.8–21.1)	1.0 (0.9–1.1)	1.0 (0.8–1.1)	2.2 (1.7–3.0)	0.4 (0.3–0.6)	0.6 (0.4–0.9)	0.5 (0.3–0.7)	1 (0.7–1.6)	0.8 (0.5–1.4)
Higher SES (n=16,209)	16.3 (15.4–17.3)	Referent	Referent	4.2 (3.7–4.7)	Referent	Referent	0.2 (0.1–0.3)	Referent	Referent
Lower SES (n=6,054)	34.9 (32.8–36.9)	2.7 (2.5–3.1)	2.1 (1.8–2.4)	6.9 (6.0–7.9)	1.7 (1.4–2.1)	1.1 (0.9–1.4)	2.1 (1.7–2.5)	10.8 (7.4–15.9)	2.1 (1.4–3.2)
Non-Native race (n=17,854)	18 (17.1–18.9)	Referent	Referent	3.7 (3.3–4.2)	Referent	Referent	0.00 (0.00–0.02)	Referent	Referent
Alaska Native race (n=4,143)	41.2 (38.7–43.7)	3.2 (2.8–3.6)	2.2 (1.9–2.6)	12.3 (11.0–13.7)	3.6 (3.0–4.3)	2.4 (2.0–3.0)	4.9 (4.1–5.7)	1172.3 (242.6–5665.2)	979 (135.9–7053.5)
Urban (n=8,908)	19.1 (17.9–20.4)	Referent	Referent	3.4 (2.9–4.0)	Referent	Referent	0.00 (0.00–0.02)	Referent	Referent
Hub (n=7,018)	22.6 (21.4–23.8)	1.2 (1.1–1.4)	1.1 (1.0–1.2)	5.4 (4.7–6.2)	1.6 (1.3–2.0)	1.4 (1.1–1.8)	0.4 (0.3–0.7)	132.4 (17.6–997.3)	56.4 (7.4–432.4)
Rural (n=6,379)	29.2 (27.8–30.7)	1.7 (1.6–1.9)	1.1 (1.0–1.3)	10.8 (9.8–11.9)	3.5 (2.8–4.2)	2.4 (2.0–3.0)	3.9 (3.3–4.6)	1217.1 (170.1–8709.3)	193.1 (26.4–1412.3)

* Model adjusted for all other demographic factors in the table.

Current SLT use prevalence was 4.9% among the total state population. Being male, younger, Alaska Native, and living in hub or rural settings were all significantly associated with odds for higher SLT use after adjustment for other factors.

Iq'mik use prevalence was only 0.7% among the total state population but 4.9% among Alaska Native adults. After adjustment for other factors, being Alaska Native and living in hub or rural communities had extremely large odds ratios for association with the current iq'mik use; being female, having children in the home, and lower SES remained significant for increased odds of iq'mik use also.

Table II describes the proportion and numbers of different demographic groups in the state's adult population, within current tobacco user populations. The highest proportions of the state's estimated 104,900 smokers were male, aged 30–49 years, had children at home, were employed, higher SES, non-Native and living in urban communities. The highest proportions of the state's estimated 23,100 SLT users were also male, aged 30–49 years, had children at home, were employed, higher SES, non-Native and living in urban communities. The state's current 3,300 iq'mik users were more often female, with children in the home, employed, lower SES, Alaska Native and living in rural settings.

**Table II:** Proportions and estimated numbers of tobacco users in Alaska in specific demographic groups

	Total Alaska adult population	Cigarette smokers	Smokeless tobacco users	Iq'mik users
	
	% population (estimated number in state)	% users (estimated number in state, rounded to nearest hundred)	% users (estimated number in state, rounded to nearest hundred)	% users (estimated number in state, rounded to Nearest hundred)
Total	100.0%	100.0%	100.0%	100.0%
		104,900	23,100	3,300
Gender: Female	48.1%	44.2%	13.4%	55.7%
		46,300	3,100	1,800
Gender: Male	51.9%	55.8%	86.6%	44.3%
		58,500	20,000	1,500
Age: 50 or better	36.3%	27.4%	20.0%	26.2%
		28,500	4,600	800
Age: 30–49	40.3%	40.1%	51.1%	45.6%
		41,700	11,700	1,500
Age: 18–29	23.4%	32.5%	28.9%	28.2%
		33,800	6,600	900
No children at home	53.5%	49.2%	40.0%	20.4%
		51,500	9,200	700
Children at home	46.5%	50.8%	60.0%	79.6%
		53,100	13,800	2,600
Currently employed	67.5%	61.6%	72.6%	48.2%
		64,200	16,500	1,500
Currently unemployed	7.3%	15.4%	15.7%	33.1%
		16,000	3,600	1,100
Currently not in workforce	25.2%	23.0%	11.7%	18.7%
		24,000	2,700	600
Higher SES	72.8%	55.7%	61.9%	20.3%
		58,300	14,300	700
Lower SES	27.3%	44.4%	38.1%	79.7%
		46,500	8,800	2,600
Non-Native race	85.6%	72.3%	64.9%	0.5%
		74,700	14,900	<100
Alaska Native race	14.4%	27.8%	35.1%	99.5%
		28,700	8,000	3,200
Urban	66.7%	59.9%	46.4%	0.3%
		62,800	10,700	<100
Hub	17.4%	18.5%	19.0%	11.0%
		19,400	4,400	400
Rural	15.9%	21.6%	34.6%	88.7%
		22,600	8,000	2,900

There were strong correlations between the 3 key variables of interest, measured using design-based Pearson Chi-square: Alaska Native race and community type (χ^2^=723.2, p<0.001), Alaska Native race and lower SES (χ^2^=689.2, p<0.001), and community type and lower SES (χ^2^=190.1, p<0.001) (data not shown).

**Fig. 1:**
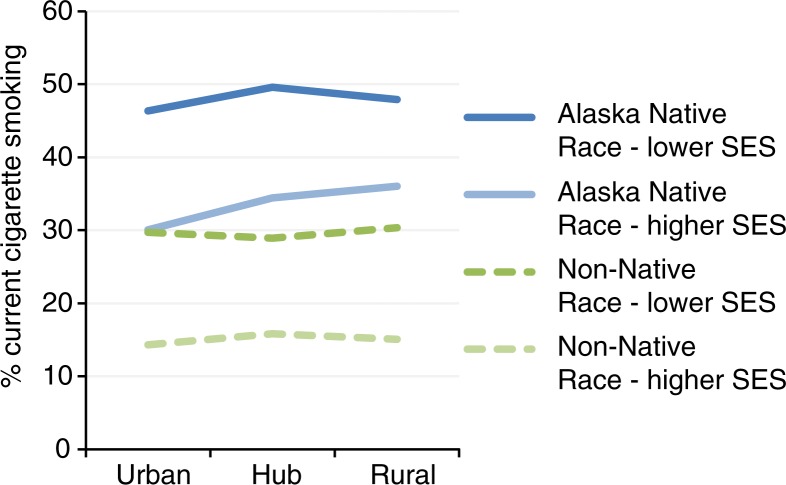
Current cigarette by population segment, Alaska BRFSS 2006–2010 combined.

**Fig. 2:**
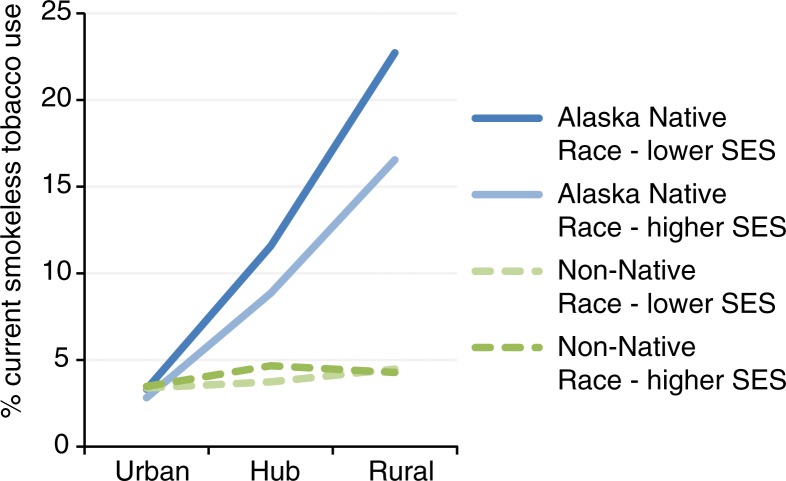
Current smokeless tobacco use by population segment, Alaska BRFSS 2006–2010 combined.

Alaska Native race, SES and community type were used to create 12 “population segments.” Tobacco use prevalence for each is displayed in Fig. 1 for cigarette smoking and Fig. 2 for SLT use. The top 3 “population segments” for each type of tobacco were identified based on: (1) prevalence of the current tobacco use; and (2) proportion of the current tobacco users (see Table III). For current smokers, non-Native urban residents (both higher and lower SES) represented the largest proportion at over 50% combined. Alaska Native, lower SES adults in all community settings, represented the highest prevalence groups, with more than 40% smoking prevalence in each group. Alaska Native, lower SES adults in rural communities were in the top tier as both highest prevalence and highest proportions of smokers. In these high-priority groups, male gender was generally dominant. Young adults were more represented in urban settings. More than two-thirds of Alaska Native smokers in these priority groups were more likely to have children in the home in all community settings, and about one-third of Alaska Native smokers in priority groups were out of work.

**Table III:** Highest proportion and highest prevalence tobacco users, with demographic characteristics

	Tobacco use		Estimated No. of tobacco users (rounded to nearest 100)	Demographic characteristics among users			
	
	% Current use (95% CI)	% of tobacco-using population		% Male	% Young adult (18–29)	% With children in home	% Out of work
Cigarette smokers							
Highest proportion							
Non-Native, high SES, urban	14.3 (13.1–15.6)	32.3	33,400	54.6	28.1	41.9	7.9
Non-Native, low SES, urban	29.7 (26.5–33.2)	19.0	19,600	55.5	42.2	55.9	23.0
Alaska Native, low SES, rural	47.9 (44.8–51.0)	9.5	9,800	62.2	34.4	69.7	34.6
Highest prevalence							
Alaska Native, low SES, hub	49.6 (44.5–54.8)	3.6	3,700	59.5	38.5	64.3	36.6
Alaska Native, low SES, rural	47.9 (44.8–51.0)	9.5	9,800	62.2	34.4	69.7	34.6
Alaska Native, low SES, urban	46.4 (37.0–56.0)	4.8	4,900	45.1	49.7	70.7	30.3
Smokeless tobacco users							
Highest proportion							
Non-Native, high SES, urban	3.5 (2.9–4.2)	34.2	7,800	98.3	26.0	49.4	3.1
Alaska Native, low SES, rural	22.7 (20.0–25.7)	18.9	4,300	59.9	28.6	82.4	40.8
Non-Native, high SES, hub	4.7 (3.9–5.6)	10.8	2,500	99.5	22.9	53.6	1.1
Highest prevalence							
Alaska Native, low SES, rural	22.7 (20.0–25.7)	18.9	4,300	59.9	28.6	82.4	40.8
Alaska Native, high SES, rural	16.5 (13.6–20.0)	7.1	1,600	64.0	39.7	70.1	27.1
Alaska Native, low SES, hub	11.6 (8.3–15.9)	3.5	800	67.6	35.3	65.6	40.8
Iqmik users							
Highest proportion and prevalence							
Alaska Native, low SES, rural	11.8 (9.9–14.2)	71.0	2,300	47.1	26.9	84.9	37.2
Alaska Native, high SES, rural	5.5 (3.9–7.7)	16.9	500	39.0	46.4	80.5	18.6
Alaska Native, low SES, hub	3.6 (2.2–6.1)	7.9	300	45.3	20.8	58.4	49.6

Similarly, for SLT users, non-Native, higher SES urban and hub residents made up the largest proportion of users (45% combined), but Alaska Native rural and lower SES hub residents had the highest prevalence of use. Alaska Native, lower SES, rural residents were among the top tier as having both the highest prevalence and proportion. Among non-Native smokeless users, nearly all were male, about half had children in the home, and very few were out of work. Among Alaska Native people who use SLT, about two-thirds were male, about one-third were young adults, more than half had children in the home, and many were out of work.

For Iq'mik users, the top tier groups of proportion and prevalence were the same: Alaska Native Rural residents in both SES categories and also Alaska Native lower-SES Hub residents. These groups were more likely to be female, often young adult, most (especially rural) users had children in the home, and many were out of work.

## Discussion

The disentangling of different populations, characteristics and approaches to prioritization is difficult. We demonstrated one approach to systematically examine data from several different perspectives: (1) a review of prevalence based on independent and adjusted factors; (2) development of “population segments” based on critical factors; and (3) prioritization of population segments separately based on prevalence and proportion, with further exploration of demographic characteristics in high-priority segments. This marketing research-like approach identified multiple high-priority population segments for Alaska's tobacco control program and provided more informative detail than describing priority populations based on single characteristics alone.

We first examined prevalence by multiple demographic factors independently, also with adjustment for other factors. This approach helped us to understand which apparently higher rates of tobacco use were potentially explained by confounding of other factors. In fact, the increased odds for cigarette smoking in hub and rural communities did not remain after adjustment for other factors. Potentially, other characteristics of the people living in more rural areas may explain the higher rates of tobacco use. Yet, the fact remains that rates are higher, regardless of why, and thus it is still important to focus specific efforts in these communities since they are unlikely to be reached effectively without specific attention.

The use of multiple key factors to describe market segments was useful and provided a clearer picture than any single factor alone. Our analyses centred on Alaska Native race, SES and community type because these were *a priori* factors that the Alaska's State Tobacco Control Program was intending to address in their program design. In general, for both cigarettes and SLT, non-Native populations that emerged as high-priority were most often urban, while Alaska Native populations were most often rural and hub. While not suggesting that these associations should be completely exclusive, the data do support developing culturally competent interventions to reach Alaska Native people in more relatively rural settings and non-Native tobacco control in more urban settings. Our findings also suggest that consideration of community setting and Alaska Native race and culture is especially important when planning SLT interventions, illustrated by the dramatic differences in prevalence associated with community type among Alaska Native people.

Generally, identifying the highest priority “population segments” using both population size- and prevalence-based approaches did not yield the same results, and provided a richer frame of consideration than either one alone. The largest proportion of both cigarette smokers and SLT users in the state was the non-Native, high SES, urban adult segment. Yet, prevalence of smoking and smokeless use was lower in this segment than most others. On the one hand, focusing efforts in low prevalence segments could be helpful if anti-tobacco community norms make the remaining tobacco users more likely to try quitting. On the other hand, some researchers have theorized that at some point of low prevalence the remaining tobacco users are highly addicted, unmotivated to change, and might be prohibitively expensive or difficult to reach effectively (16). If this occurs, there might be more benefit to deploying resources in higher prevalence communities with fewer, but more motivated tobacco users. Perceived receptivity could be assessed in program planning, for example, by examining recent attempts to quit smoking in the different population segments.

Providing demographic descriptions of the high-priority groups was useful in further thinking about development of salient messages and interventions. We saw that Alaska Native people were consistently more likely than non-Native people to have children in the home; therefore, the data suggest that tobacco control efforts tailored to serve Alaska Native people and their families may reach young people, including for tobacco prevention, and when considering this secondary audience the potential benefits of Alaska Native-specific efforts become even greater. In particular, having children in the home was independently associated with SLT use, and this information may be useful for tailoring tobacco control messages, or possibly deserves more study to understand motivations behind use of SLT, such as if adults are switching from smoking to SLT to protect children from second-hand smoke. Also, the high percentage of Alaska Native SLT users who are out of work and very low percentage of non-Native SLT users who are out of work provides input for design of campaigns to reach either group.

Given that resources are generally limited, a focus on broad population-based strategies (including taxes, second-hand smoke bans) that reach all populations is efficient. But within these interventions, attention can be given to tailoring messages and supportive services so that they reach and are effective with high-priority population segments in an appropriate way.

Alaska is addressing this by offering community-based grant applicants a set of interventions that show population-wide impact, but also may be uniquely deployed regionally, and asking grantees to describe how they will specifically reach Alaska Native and low SES people, including through systems change in the organizations or structures that serve them.

We were able to use the existing surveillance data to apply market research-like approaches without additional costs of data collection. This was an efficient strategy for better understanding populations to serve, although multiple years of data were required to generate sufficient numbers of respondents for robust analyses. However, even if more data were available, quantitative analyses alone cannot provide definitive answers about which people need help the most, and how to reach them. This analysis is an example of providing data in a way that more meaningfully informs the discussion and thinking of program leaders and stakeholders – one that can be used by them in combination with other considerations to provide greater opportunities for programmatic successes. Additionally, trends in health factors can be monitored for specific segments, to evaluate the effectiveness of public health efforts.

### Limitations

Several limitations apply to our study. First, the Alaska BRFSS is limited to adults living in households with landline telephones and who speak English. These respondents may not be representative of the entire population. Second, our classification of current tobacco users did not include frequency, therefore some people that we classified as “current users” could potentially be infrequent or inconsistent smokers or SLT users. Third, some measures that we used to describe individuals (such as SES and urban–hub–rural community residence) may misclassify our intended factors – having insufficient access to resources and living in increasingly remote locations.

## Conclusions

While Alaska Native race, low SES status and community of residence can each be considered alone, and tobacco control interventions planned accordingly, our study showed that thinking of “population segments” may be helpful for better tailoring effective tobacco control interventions. The identification of high-priority population segments based both on prevalence and proportion of tobacco use, with subsequent description by demographic factors, may be useful for program design in countries or regions with mixed community contexts and indigenous groups.

## References

[1] Centers for Disease Control and Prevention (2012). World No Tobacco Day – May 31, 2012. MMWR Morb Mortal Wkly Rep.

[2] US Department of Health and Human Services (2010). How tobacco smoke causes disease: the biology and behavioral basis for smoking-attributable disease: a report of the Surgeon General.

[3] Chateau-Degat ML, Dewailly E, Louchini R, Counil E, Noël M, Ferland A (2010). Cardiovascular burden and related risk factors among Nunavik (Quebec) Inuit: insights from baseline findings in the circumpolar Inuit health in transition cohort study. Can J Cardiol.

[4] Egeland GM, Cao Z, Young TK (2011). Hypertriglyceridemic-waist phenotype and glucose intolerance among Canadian Inuit: the International Polar Year Inuit Health Survey for Adults 2007–2008. CMAJ.

[5] Alaska Department of Health and Social Services (2007). What state surveys tell us about tobacco use among Alaska Natives: implications for program planning.

[6] United States Federal Trade Commission Cigarette report for 2009 and 2010. http://ftc.gov/os/2012/09/120921cigarettereport.pdf.

[7] FTC Report Shows Big Decline in Cigarette Sales after 2009 Federal Cigarette Tax Increase, While Tobacco Companies Still Spend Huge Sums on Marketing (2012). Campaign for Tobacco-Free Kids. Statement of Susan M. Liss, Executive Director.

[8] Haire M (1950). Projective techniques in marketing research. J Market.

[9] Dowling GR (2004). Market segmentation and targeting.

[10] Tobacco Prevention and Control Program (2011). A decade of progress: tobacco prevention and control in Alaska FY 2010–2011.

[11] Tobacco Prevention and Control Program (2011). Alaska strategic plan for eliminating tobacco-related disparities – 2011 update.

[12] Alaska Department of Health and Social Services (2012). Tobacco in the Great Land, a portrait of Alaska's leading cause of death, 2012 update.

[13] State of Alaska Department of Labor and Workforce Development (2013). Alaska economic trends. http://labor.alaska.gov/trends/apr13.pdf.

[14] Alaska Department of Health and Social Services Alaska's behavioral risk factor surveillance system. State of Alaska.

[15] Beltz DN (1996). Tobacco use in rural Alaska and the trampling tobacco project. Alaska Med.

[16] Warner KE, Burns DM (2003). Hardening and the hard-core smoker: concepts, evidence and implications. Nicotine Tob Res.

